# Teigne favique traitée par la terbinafine chez un enfant de 11 ans

**DOI:** 10.11604/pamj.2015.22.368.6705

**Published:** 2015-12-14

**Authors:** Khalid Lahmadi, Mohamed Elhaouri

**Affiliations:** 1 Laboratoire de Biologie de l'Hôpital Militaire Moulay Ismail de Meknès, Meknès, Maroc; 2 Service de Dermatologie de l'Hôpital Militaire Moulay Ismail de Meknès, Meknès, Maroc

**Keywords:** Favus, terbinafine, Trichophyton schoenleini, griséofulvine, Favus, terbinafine, Trichophyton schoenleini, griseofulvin

## Image en medicine

Patient âgé de 11 ans qui s'est présenté avec des lésions étendues du cuir chevelu. Au milieu de ces lésions, les cheveux étaient ternes. A leur base se trouvait une dépression circulaire recouverte d'une masse croûteuse, de couleur jaunâtre (A). La lésion dégageait une odeur nauséabonde. L'examen mycologique des cheveux atteints était positif. La culturesur milieu Sabouraud chloramphénicol et Sabouraud chloramphénicol + actidione a permis l'isolement Trichophyton schoenleinii (B). Le diagnostic d'une teigne faviquea été retenu. L'enfant a été mis sous terbinafine 125mg/jour en une seule prise quotidienne par voie orale pendant quatre semaines associée à un traitement local par l'econazole 1% crème en deux applications par jour. L’évolution a été marquée par la disparition des lésions inflammatoires et persistance des plaques alopéciques (C, D). La terbinafine est un antifongique qui commence de plus en plus à être utilisé pour le traitement des teignes du cuir chevelu. Son AMM a été obtenu pour les patients âgés de plus de 15 ans. Cependant, certaines études ont montré une bonne tolérance de ce médicament chez des enfants moins âgés. La terbinafine, utilisée à la dose de 4 à 5mg/kg/jour en une prise quotidienne par voie orale pendant une durée moyenne de quatre semaines, serait plus efficace que la griséofulvine à la dose de 20 à 25mg/kg/jour en deux prises pendant 6 à 8 semaines sur les teignes du cuir chevelu dues aux champignons du genre Trichophyton. La terbinafine s'est révélé efficace chez notre patient.

**Figure 1:**
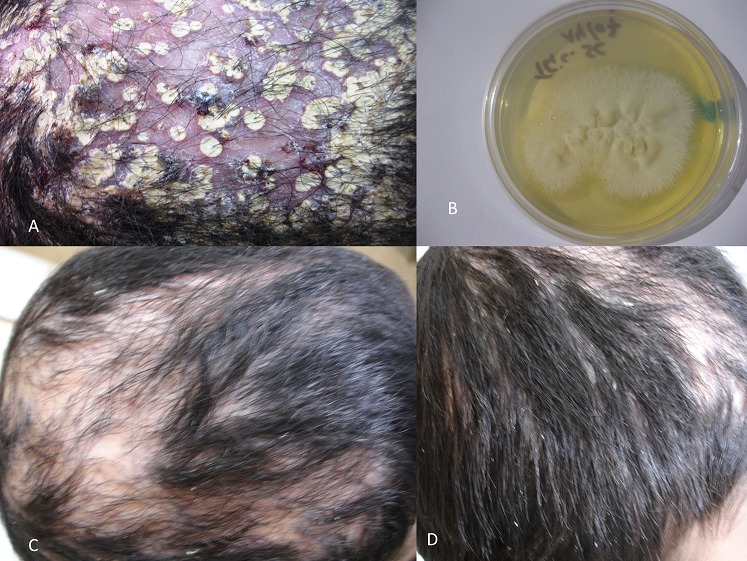
A) teigne favique (GODET FAVIQUE); B) colonie de Trichophyton schoenleinii en culture sur milieu Sabouraud chloramphénicol; C,D) évolution après traitement

